# The Role of Toxins in the Pursuit for Novel Analgesics

**DOI:** 10.3390/toxins11020131

**Published:** 2019-02-23

**Authors:** Yossi Maatuf, Matan Geron, Avi Priel

**Affiliations:** The Institute for Drug Research (IDR), School of Pharmacy, Faculty of Medicine, The Hebrew University of Jerusalem, Jerusalem 9112001, Israel; yossef.maatuf@mail.huji.ac.il (Y.M.); matan.geron@mail.huji.ac.il (M.G.)

**Keywords:** TRPV1, TRPA1, ASIC, Na_V_ channels, chronic pain, analgesics, toxins, pharmacophore.

## Abstract

Chronic pain is a major medical issue which reduces the quality of life of millions and inflicts a significant burden on health authorities worldwide. Currently, management of chronic pain includes first-line pharmacological therapies that are inadequately effective, as in just a portion of patients pain relief is obtained. Furthermore, most analgesics in use produce severe or intolerable adverse effects that impose dose restrictions and reduce compliance. As the majority of analgesic agents act on the central nervous system (CNS), it is possible that blocking pain at its source by targeting nociceptors would prove more efficient with minimal CNS-related side effects. The development of such analgesics requires the identification of appropriate molecular targets and thorough understanding of their structural and functional features. To this end, plant and animal toxins can be employed as they affect ion channels with high potency and selectivity. Moreover, elucidation of the toxin-bound ion channel structure could generate pharmacophores for rational drug design while favorable safety and analgesic profiles could highlight toxins as leads or even as valuable therapeutic compounds themselves. Here, we discuss the use of plant and animal toxins in the characterization of peripherally expressed ion channels which are implicated in pain.

## 1. Introduction

Pain is a physiologically important phenomenon as it alerts an organism to tissue damage or potential tissue damage [1]. Pain is initiated when peripheral terminals of a subgroup of sensory neurons, termed nociceptors, are activated to produce action potentials [2]. This depolarization of nociceptors is produced by specialized pain receptors that detect various chemical, thermal, and mechanical noxious stimuli [2,3]. The pain signal is then transmitted to the spinal cord dorsal horn and eventually to higher regions in the central nervous system (CNS) where it is processed [4]. Subsequently, an appropriate response to the noxious stimulus is generated to avoid further injury [1,5]. Moreover, the memory of pain deters the affected organism from repeating actions that evoke this unpleasant experience [6].

However, this pain sensation following exposure to noxious stimuli (i.e., acute pain) could be undesirable when undergoing a medical procedure or when the pain is too intense and debilitating following injury [7]. Chronic pain is another instance in which suppression of the nociceptive system is required. Chronic pain is defined as a sensation of pain that persists long after the expected healing of the underlying injury when pain is no longer serving any useful role [8,9,10]. Indeed, chronic pain is among the leading causes of seeking medical attention, accountable for about 20% of patients in primary care [11]. This pain pathology can stem from nerve damage (neuropathic pain) or be associated with conditions that produce continuous stimulation of the pain pathway, such as inflammation [8,9,12]. Chronic pain can be accompanied by plastic changes to nerves leading to altered detection, transmission, processing, and regulation of pain [13,14]. These impairments generate an abnormal and hyperexcitable function of the nociceptive system, leading to persistent and intensified pain sensations [13].

Currently, treatment of chronic pain is lacking as the available drugs achieve only partial analgesia and in just a fraction of the patients [14]. To date, most analgesics in use target ion channels and receptors in the spinal cord and brain. Thus, these agents modulate the transmission and processing of the pain signal centrally [15]. Additionally, the targets of these drugs are involved in processes other than nociception [16]. For example, opiates provide varying degrees of efficacy in the treatment of different pain types by activating opioid receptors in spinal and supra-spinal domains [13]. Due to their central activity, these agents are notorious in producing serious adverse effects, including respiratory depression, sedation, euphoria, dependence, and addiction [13,16]. While opiates also produce peripheral unwanted effects, these CNS-related side effects are especially concerning as opioid abuse and opioid-related deaths have gained epidemic proportions in the United States. Thus, pain pathologies in which opiates are also moderately effective (e.g., neuropathic pain) are preferably treated with atypical analgesics (e.g., pregabalin, duloxetine, amitriptyline) [14]. However, most of these analgesics commonly in use were initially developed and are prescribed for the treatment of other diseases such as depression and epilepsy while also presenting poor selectivity to their targets in some cases [14,15,17]. Due to this, patients experience numerous side effects when treated with these agents [16]. These adverse effects may reduce the compliance to the pharmacological therapy and further contribute to the failure of pain management. Thus, there is a dire need for novel, safe, and efficacious analgesics for the treatment of chronic pain. 

Receptors and ion channels in the peripheral terminals and axons of nociceptors were shown to be pivotal in the generation of pain [1,2,18]. It is possible that more effective analgesia could be achieved by targeting transduction and transmission in nociceptors, thus blocking pain at its source [19]. Additionally, analgesics with a peripheral site of action can exert an improved safety profile. This can be achieved by targeting proteins that are expressed selectively in nociceptors [16]. Another avenue is to design agents that cannot penetrate the blood-brain barrier into the CNS. Indeed, there is a growing effort in the search for new analgesics that act peripherally [20]. Transient receptor potential vanilloid 1 (TRPV1) and transient receptor potential ankyrin 1 (TRPA1) are two pain receptors that emerged as potential targets for such analgesics [20]. These cation channels are activated by numerous noxious stimuli from many sources including inflammatory mediators and were suggested to have a role in the detection of noxious temperature [19]. As pain pathologies often involve altered sensitivity to heat or cold, suppressing TRPV1 and TRPA1 activation could be a promising approach [20]. Acid-sensing ion channels (ASICs) are another pain receptors that are drawing attention in this context as these cation channels (with high preference to sodium) were shown to be involved in inflammatory pain and chronic pain conditions [21,22]. Blocking the action potential propagation through the nociceptor axon by modulating voltage-gated sodium channels (Na_V_) could also be highly effective in alleviating pain [23]. It was found that several Na_V_ channels are important in evoking action potentials in nociceptors where they are selectively expressed [24]. Thus, specific attenuation of the pain signal could be obtained by inhibiting these channels. 

The development of new modulators requires deep understanding of both the structure and function of ion channels. Natural toxins can be used to gain such insights as they affect functionally essential domains in ion channels [6,25]. Additionally, toxins have evolved to be stable, potent and specific to proteins that are physiologically significant [6,26]. These features highlight the importance of toxins in identifying new targets for pharmacological intervention and in the design of novel drugs. Moreover, toxins can be used as lead compounds in the process of drug development or as drugs themselves [21,27]. A striking example for this is ziconotide, a synthetic version of a toxin found in the venom of the cone snail *Conus magus*, which was approved by the FDA in 2004 for the treatment of severe refractory chronic pain [13]. Due to the peptidic nature of this toxin, it has to be injected intrathecally where it inhibits the N-type voltage-gated calcium channels through binding to their α_1B_ subunit [13,28,29]. By inhibiting these pre-synaptic channels in the central terminals of nociceptors, ziconotide reduces the release of pro-nociceptive neurotransmitters thereby disrupting the transmission of pain signals in the spinal cord [13]. As evoking an aversive response could be a useful tool in the defensive arsenal of venomous organisms, the pharmacopeia libraries that are venoms contain numerous toxins known to modulate nociceptive targets and probably many more such toxins that are yet to be identified. Undeniably, these toxins were and will be instrumental in understanding the nociceptive system.

**Figure 1:**
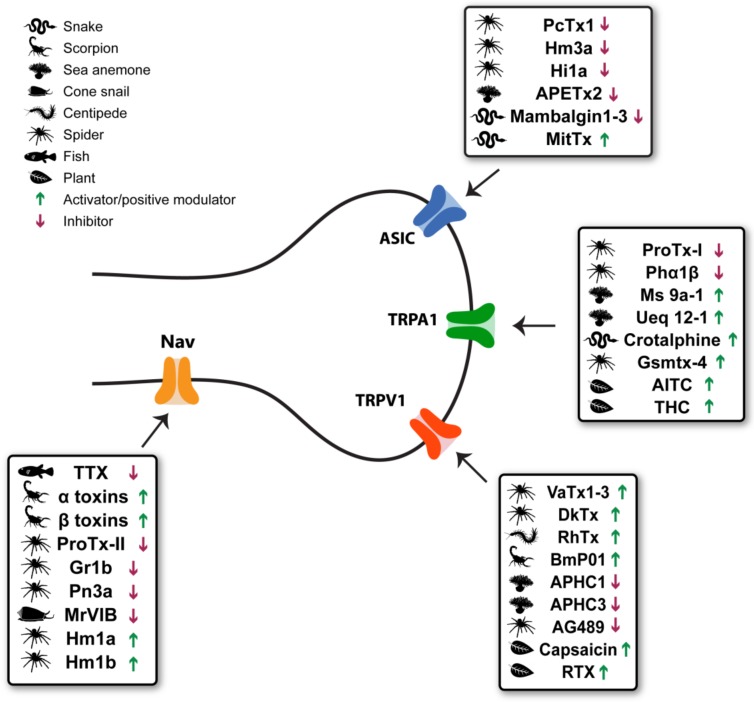
Schematic representation of plant and animal toxins targeting ion channels involved in pain. The following represents only a partial list of toxins that have been found to modulate the activity of TRPV1, TRPA1, ASIC, and Na_V_ channels.

In this review, we will focus on plant and animal toxins targeting the aforementioned prominent ion channels that are peripherally expressed in nociceptors (Figure 1). We will evaluate the contribution of these toxins to the study of the structure and function of these channels. Additionally, toxins’ potential role in the design of novel ion channel modulators aiming at analgesia will be discussed.

## 2. Transient Receptor Potential Vanilloid 1 (TRPV1)

TRPV1 is a non-selective cation channel (with preference to Ca^2+^) that is predominantly expressed in the axons and in both the peripheral and central terminals of nociceptors [2,30]. This pain receptor assembles as a homo-tetramer where its four subunits are arranged around an ion permeable pore [31]. Each subunit has intracellular N- and C-terminals and six transmembrane segments (S1–S6). S5 and S6 along with the pore helix that resides between them line the pore [31]. TRPV1 is part of the transient receptor potential (TRP) family consisting of 28 different channels in mammals that share similar topology [32]. Based on sequence homology TRPV1 can be further sub-classified as a member of the transient receptor potential vanilloid family (TRPV) [33]. 

TRPV1 is also a part of 10 TRP channels (TRPV1-4, TRPM2, TRPM3, TRPM5, TRPM8, TRPC5, and TRPA1) that are activated by temperature, termed thermoTRPs [34]. As each of these channels responds to a specific range of temperatures, TRPV1 is activated by heat (≥43°) [30,35]. Other activators of the multi-steric TRPV1 include protons and bioactive lipids like anandamide, N-arachidonoyldopamine (NADA), and lipoxygenase products, which are mainly released in inflamed tissues [2,36,37]. However, while the binding sites of these molecules were previously defined, the TRPV1 domains that are important for activation by heat were not thoroughly characterized [38]. TRPV1 is also activated and sensitized indirectly by a wide variety of other inflammatory mediators such as histamine, bradykinin, prostaglandins, and ATP, as well as NGF, which also upregulates TRPV1 expression [39,40,41]. When bound to their receptors on nociceptor terminals, these algogens trigger the activity of the phospholipase C (PLC) signaling pathway. This leads to the phosphorylation and sensitization of TRPV1 by protein kinase Cε (PKCε) and the production of PIP2 derivatives, which further contribute to the channel’s activation [2,32]. TRPV1 is also phosphorylated and sensitized by protein kinase A (PKA) [42]. Sensitization of TRPV1 can result in lowered heat activation threshold, producing continuous activation at body temperature levels [43]. Thus, overall, the polymodal TRPV1 is an essential integrator of pain signals from many sources. Additionally, TRPV1 is often expressed in peptidergic nociceptors in which depolarization evokes the antidromic secretion of calcitonin gene-related peptide (CGRP) and substance P. As these neuropeptides promote neurogenic inflammation, vasodilation and edema, TRPV1 activation may further enhance inflammatory processes in the tissue in a positive feedback loop [2,44]. In addition, TRPV1 expressed pre-synaptically in central terminals of nociceptors is involved in pain signal transmission by promoting the release of excitatory neurotransmitters like CGRP, glutamate, and substance P in the spinal cord [15,45].

TRPV1 is implicated in several pain conditions. Knockout experiments showed that TRPV1 is necessary for heat hyperalgesia, which is a common symptom in chronic pain pathologies [15,46]. Noxious heat sensitivity was also suggested to be impaired in these mice [47]. In accordance, ablation of TRPV1-expressing nociceptors using diphtheria toxin eliminated pain heat sensitivity [48,49]. Additionally, TRPV1 was shown to have a role in maintaining ectopic firing and peripheral and central sensitizations [45,50,51]. Expression of TRPV1 can vary in different pain syndromes. It was found that TRPV1 is upregulated in post-herpetic neuralgia, bone cancer and in inflammation in which the channel might even be expressed in sensory neurons that do not usually express TRPV1 [19]. In contrast, channel expression was found to be reduced in some neuropathic pain models [19,52]. However, the clinical relevance of these changes in channel expression remain unclear. In addition, TRPV1 was associated with several visceral pain conditions by using channel inhibitors, knockout experiments and examining the channel’s expression [4,45,53,54]. Nonetheless, the exact mechanism of TRPV1 involvement in visceral pain pathologies still remains unclear in most cases. Overall, a role of TRPV1 was demonstrated in diabetic neuropathy, post-herpetic neuralgia, postsurgical neuropathic pain, complex regional pain syndromes, peripheral neuropathic pain, bone cancer pain, chronic inflammatory pain, irritable bowel syndrome, bladder cystitis, rheumatoid arthritis, and osteoarthritis [4,45,54,55,56]. Thus, its physiological and pathophysiological properties make TRPV1 an attractive target for the development of new analgesics.

Due to its important role in evoking pain and eliciting an aversive response, TRPV1 also serves as a target for various plant and animal toxins (Table 1) [6,57]. Capsaicin, the pungent ingredient in chili peppers, is considered the prototypical TRPV1 activator and is used extensively to investigate the channel’s properties. In fact, this potent vanilloid molecule (EC_50_ = 50–200 nM: HEK293 cells) was employed in the cloning of TRPV1 [30]. Capsaicin was shown to activate TRPV1 through the vanilloid binding site (VBS) found in the intracellular side of the channel between S3 and S4 [31,58]. The VBS was also identified as the binding site of endogenous bioactive lipids that share structural similarity with capsaicin [2,58]. Resiniferatoxin (RTX) from the *Euphorbia resinifera* cactus is another plant toxin that binds to the VBS and activates TRPV1 [59,60]. RTX is an ultra-potent activator with an EC_50_ at least ten-fold smaller than capsaicin’s (EC_50_ = 0.3–11 nM: HEK293 cells) [49,61,62]. By configuring their binding orientation, both RTX and capsaicin significantly contributed to the understanding of the VBS dynamic architecture and its coupling to the TRPV1 pore, which allows channel activation [63,64]. Due to the robust Ca^2+^ influx that capsaicin and RTX evoke, they are also used for activating or ablating TRPV1 expressing cells, thus providing insights to the role of these specific nociceptors in somatic or visceral pain sensations [4,15,65,66]. Due to the involvement of TRPV1 in visceral pain, capsaicin is also used in order to produce a much needed animal model of abdominal pain. Indeed, intracolonic injection of capsaicin evokes abdominal mechanical hyperalgesia and abdominal pain related behavior [45,67]. Thus, capsaicin greatly contributed to the understanding of the mechanisms that underlie this widespread visceral pain condition. 

The first animal toxins that were found to activate TRPV1 are vanilotoxins 1–3 (VaTx1–3) [68]. These three peptides, derived from the venom of the tarantula *Psalmopoeus cambridgei*, were shown to bind the channel’s outer pore region [68]. Thus, VaTx1–3 demonstrated the role of this structural domain in channel gating and highlighted it as a possible target for novel TRPV1 modulators. Another spider toxin, the double-knot toxin (DkTx) is a unique TRPV1 activating toxin found in the venom of the Earth tiger tarantula (*Ornithoctonus huwena*) [69]. DkTx is a peptide toxin consisting of two inhibitory cystine knot (ICK) motifs connected by a linker. As revealed in both functional and structural studies, this toxin binds to the outer pore region of the channel where the two knots of one DkTx molecule bind simultaneously to adjacent subunits in the TRPV1 tetramer [69,70,71]. Its exceptional bivalent interaction allows DkTx to bind irreversibly to the channel and lock it in an open state. As a result, DkTx evokes persistent non-washable activation of TRPV1 that is presumed to account for the intense and prolonged pain associated with the spider bite [69]. DkTx ability to produce stable activation of TRPV1 proved decisive in capturing the channel in an open state using cryo-EM [72,73]. This complex, that also included RTX, demonstrated the widening of both the selectivity filter and the lower gate in the TRPV1 pore [73]. DkTx along with capsaicin further demonstrated the importance of the TRPV1 pore turret in channel gating [74]. This structural domain was found to restrict widening of the outer vestibule of the pore when DkTx is bound while stabilizing the open state in capsaicin-activated channels [74]. Thus, DkTx has served as an essential tool in elucidating TRPV1 activation mechanism and structure. These advancements among others in structural biology significantly contribute to the effort of rationally designing new TRPV1 modulators by producing relevant pharmacophores. Additionally, the bivalency, which is responsible for the increased potency (EC_50_ = 0.23 µM; HEK293 cells) and irreversible binding of DkTx could represent a viable approach in the design of new long-acting modulators of TRPV1 [69]. However, the effect of DkTx itself on pain sensation and nociceptors was not yet tested. 

Other toxins that promote TRPV1 activation are found in centipede (RhTx) and scorpion (BmP01) venoms [75,76]. The modulation mechanism of TRPV1 by these peptides is related to other modalities that activate this channel. RhTx-induced activation was shown to be heat-dependent as the toxin activity is reduced in lower temperatures. Furthermore, low RhTx concentrations potentiate TRPV1 response to heat [75]. As mutagenesis experiments placed the RhTx binding site in the outer pore region, it was suggested that this toxin could shed more light on the heat sensing machinery of TRPV1 [57,75]. BmP01 activity, on the other hand, is associated with the gating mechanism induced by protons [77]. The response to this scorpion toxin is potentiated by acidic pH while in turn the toxin itself potentiates protons-induced activation. Additionally, it was shown that a residue in the outer pore region of TRPV1 is crucial for channel activation by both BmP01 and protons, further establishing a connection between the activation mechanisms of these two modalities [77]. While both RhTx and BmP01 produce pain when injected to mice, these toxins present the possibility of developing TRPV1 modulators that produce fine-tuning of the channel activation by physiological stimuli. This may prove useful in evoking a desirable response while preventing unwanted side effects. 

Perhaps counter-intuitively, TRPV1 activating toxins are also used as analgesics. Indeed, topical treatments of capsaicin can promote pain relief in certain pain conditions with formulations of low-dose (0.075%) creams and high-dose (8%) patches currently in use [78,79]. The mechanism of action by which this phytotoxin promotes analgesia is not entirely understood [45]. However, it has been suggested that following an initial burning sensation, capsaicin induces Ca^2+^-dependent desensitization of TRPV1 rendering it insensitive to capsaicin as well as to other stimuli [20,79]. In addition, it was suggested that topical capsaicin promotes the depletion of pro-inflammatory neuropeptides from TRPV1-expressing nociceptor terminals [45,79,80]. High dose or repeated applications may also evoke robust activation of TRPV1, thus allowing a massive influx of Ca^2+^ ions [45,81]. As a result, other channels in the affected nociceptor (e.g., P2X_3_, TRPV2, and TRPA1) can be desensitized and inhibited while affected axons might undergo reversible degeneration altogether [45,82]. Thus, this defunctionalization of the TRPV1-expressing neurons, which are polymodal, also blocks the transduction of noxious stimuli that do not necessarily affect TRPV1. Due to its high potency, RTX is considered for the treatment of severe pain in patients with advanced cancer in palliative care. Pain relief in these patients is achieved by intrathecal injections of RTX and ablation of the central terminals of TRPV1- expressing nociceptors in the dorsal horn [65]. Capsaicin can also be used to facilitate the delivery of other drugs. As TRPV1 is permeable to large cations, activation of the channel by capsaicin enables the charged anesthetic QX-314 to cross the membrane of nociceptors [23,83]. Intracellularly-trapped QX-314 can then block voltage-gated sodium channels and produce long-lasting elimination of pain signal transmission [23,83]. Thus, co-application of capsaicin and QX-314 specifically silences TRPV1-expressing nociceptors. 

TRPV1 antagonists were long considered as potentially promising analgesics. Indeed, while many TRPV1 antagonists were not found to be beneficial, others proved effective in reducing pain in certain nociceptive and neuropathic pain models including bone cancer pain and osteoarthritis [49,84,85,86]. However, the main obstacle for introducing most of these agents to the clinic remains their unsatisfactory safety profile [87]. Since TRPV1 antagonists heighten the noxious heat threshold substantially, patients are in increased risk of a scalding injury [78]. Another serious side effect associated with antagonists is hyperthermia [88]. Both pre-clinical and clinical trials demonstrated that subjects might develop an elevated core body temperature when treated with TRPV1 antagonists [87]. These results imply that TRPV1 is involved in central thermal regulation. A few suggestions were raised in an attempt to tackle these on-target side effects. According to one approach, modality-specific antagonists that inhibit TRPV1 activation by capsaicin and inflammatory mediators, but spare the heat-induced activation, could prevent these temperature-related adverse effects [78]. However, while TRPV1 is multi-steric, the lack of knowledge regarding the channel domains that are important for heat sensation makes such rational design a difficult task. Another avenue could be the design of use-dependent antagonists that bind open/desensitized channels, thus inhibiting only hypersensitive TRPV1 channels and not those that are activated physiologically. 

Although their evolutionary benefit is not yet understood, toxins that inhibit TRPV1 were also found. Such toxins are the peptides APHC1 and APHC3 from the venom of the sea anemone *Heteractis crispa* [55,89]. Molecular modeling analysis suggests that APHC1 and APHC3 bind to the outer pore region of TRPV1, illustrating the possibility of antagonizing the channel through this domain [89]. In vitro studies showed that these toxins exhibit a bi-modal effect. While APHC1 and APHC3 were shown to partially inhibit the response to high capsaicin concentrations, these toxins also potentiated TRPV1 activation by low concentrations of capsaicin and protons [90]. Both toxins showed analgesic effects in acute and chronic pain models in mice without causing hyperthermia [55]. Thus, APHC1 and APHC3 demonstrate that partial inhibition or mixed potentiation/inhibition effect on TRPV1 might prevent this side effect. Another toxin that was found to antagonize TRPV1 is AG489 [91]. This polyamine toxin derived from the venom of the spider *Agelenopsis aperta* was suggested to occlude the channel’s pore [91]. However, AG489 is not selective as it blocks ASIC and NMDA channels as well [91].

## 3. Transient Receptor Potential Ankyrin 1 (TRPA1)

Another member of the TRP ion channel family is the ankyrin-type, known as the TRPA subfamily. So far, the only member of the TRPA subfamily identified in mammals is the TRPA1 channel [104,105]. TRPA1 is a non-selective cation channel that exhibits a high preference for calcium ions. Like other TRP family members, four TRPA1 subunits assemble to form a functional channel. Each subunit is composed of six transmembrane helices (S1–S6) and cytoplasmic N- and C- termini. The S1–S4 helices form the gating sensor domains, while the pore domain is formed by the S5 and S6 segments. A unique feature that distinguishes TRPA1 from other TRP channels is an exceptionally long region within the N-terminus containing up to 18 ankyrin repeat domains in humans. Ankyrin repeats are known protein-protein interacting domains, which also could be essential for channel regulation and plasma membrane localization [106,107,108]. TRPA1 is predominantly co-expressed with TRPV1 channels, in non-myelinated C fibers of trigeminal and dorsal root ganglia neurons. This subset of primary sensory neurons is known to mediate irritant effects and inflammatory pain [109,110]. In line with this, TRPA1 is activated by various irritant electrophilic and non-electrophilic compounds, which can elicit pain in animals and humans. For example, agents such as allyl isothiocyanate (AITC) from mustard oil, cinnamaldehyde from cinnamon, and allicin from garlic are highly reactive electrophiles that activate TRPA1. These compounds activate the TRPA1 receptor through covalent association with cysteine residues within the cytoplasmic N terminus, causing a conformational change that opens the channel [111,112,113]. As mentioned, TRPA1 can also be activated by many non-electrophilic compounds such as menthol, carvacrol, thymol, and Δ^9^-tetrahydrocannabinol (THC) [114,115,116,117]. Unlike electrophilic compounds, non-electrophilic agents do not interact with the cysteine residues in the N-terminus of the channel, suggesting the existence of additional selective binding sites. However, the activation mechanisms for non-electrophilic ligands are still elusive [113,118]. Moreover, several endogenous agonists that are generated under various pathophysiological conditions, such as tissue injury and inflammation, have been found to modulate TRPA1 activity. Several lines of evidence suggest that the activation of TRPA1 by endogenous agonists plays a critical role in the pathogenesis of pain and inflammation [119,120,121,122,123]. It has been postulated that TRPA1 contributes not only to acute pain sensation, but may also be involved in the process of transition from acute to chronic pain [123,124,125,126]. Furthermore, the role of TRPA1 in visceral hypersensitivity has been thoroughly studied [127,128]. TRPA1 is expressed in visceral afferent sensory neurons and appear to play a major role in visceral inflammation and nociception [129,130,131,132,133,134,135]. In fact, administration of TRPA1 agonists, such as mustard oil, are widely considered as models of visceral pain in rodents [128]. Based on the concept that TRPA1 is active during pathological conditions, TRPA1 antagonists have been actively pursued [136,137,138,139,140]. Although inhibition of TRPA1 appears to be the most logical therapeutic strategy for neuropathic pain management, several research groups have demostrated that TRPA1 agonists may produce analgesia [129,141,142,143,144]. TRPA1 agonists most probably attenuate pain sensation and inflammatory responses via desensitization of sensory neurons expressing TRPA1, analogous to capsaicin desensitization of TRPV1-expressing neurons. The exact mechanism underlying the analgesic and anti-inflammatory effects of TRPA1 agonists remains to be elucidated. 

To date, a number of toxins have been shown to modulate the TRPA1 receptor (Table 2). One such toxin is Protoxin I (ProTx-I) that was isolated from the venom of Peruvian green velvet tarantula (*Thrixopelma pruriens*). Previously identified as an antagonist of voltage-gated sodium channels, this 35-residue peptide was recently shown to also antagonize the TRPA1 receptor with high affinity [145,146,147]. Further analysis revealed that ProTx-I inhibits both types of channels by binding to the extracellular loops of the S1–S4 domains. Based on the structure of ProTx-I, a mutant peptide was engineered which was the first effective antagonist that only affects TRPA1 without disrupting the activity of other ion channels [147]. These findings open the possibility of using this peptide as a lead in the development of new TRPA1 blockers. Furthermore, by configuring its binding site, ProTx-I greatly contributed to the understanding of TRPA1 gating mechanism which may contribute to the effort of rationally designing new TRPA1 modulators. Similarly, the toxin Phα1β, which was purified from the venom of the Brazilian armed spider (*Phoneutria nigriventer*), is a selective TRPA1 receptor antagonist that does not interact with other TRP channels [148]. Previous studies demonstrated that both acute and chronic pain could be reduced by administration of Phα1β in several animal pain models. These findings suggest that this toxin may potentially be used as a therapeutic agent for the management of inflammatory and neuropathic pain [149,150,151,152,153]. However, it is important to note that Phα1β was also found to inhibit voltage-gated calcium channels (VGCC) as intra- and extracellular calcium ions play a major role in regulating the activity of TRPA1 channels [150,154,155,156]. It has been speculated that Phα1β has a distinct analgesic mechanism of action in different pain conditions. In post-operative pain model, Phα1β may induce analgesic effects via inhibition of VGCC, whereas in chemotherapy-induced peripheral neuropathy models Phα1β exert its therapeutic activity through the inhibition of TRPA1 [148]. Thus, Phα1β may represent a potential novel lead compound with distinct action mechanisms in different pain disorders. Further research is needed to investigate the dual activity of Phα1β on both of the TRPA1 channels and VGCC and its relevance in various pain states.

The toxin peptides τ-AnmTX Ms 9a-1 (Ms 9a-1) and τ-AnmTX Ueq 12-1 (Ueq 12-1), from the venom of the sea anemone *Metridium senile*, act as positive modulators of TRPA1 in vitro [144]. Application of these peptides alone did not induce any significant activation of TRPA1, but they potentiate the activation of TRPA1 induced by different agonists. Interestingly, when injected into mice, Ms 9a-1 and Ueq 12-1 produce significant analgesic and anti-inflammatory effects. The authors suggested that the toxins produce the significant analgesic effect in vivo through desensitization of the TRPA1 receptor. According to this hypothesis, Ms 9a-1 and Ueq 12-1 potentiate the response of TRPA1 to endogenous agonists, which results in weak but sustained activation of the receptor leading to functional loss of TRPA1-expressing neurons [144,157]. These toxins demonstrate the possibility of selectively silencing only TRPA1 channels that are active by potentiating their response to other activators. This approach may facilitate the development of TRPA1-targeting analgesics with an improved safety profile.

Another toxin that modulates TRPA1 activity is crotalphine [158]. Crotalphine is a 14 amino acid peptide that was first isolated from the venom of the South American rattlesnake (*Crotalus durissus terrificus*). Previous works demonstrated that crotalphine, when administered in vivo, induces potent and long-lasting (3–5 days) analgesic effects in acute and chronic pain models. It has been proposed that the anti-nociception induced by crotalphine is related to the activation of peripheral opioid receptors [159,160,161,162]. However, despite presenting opioid activity, crotalphine does not directly bind to opioid receptors [158,161]. Thus, it appears that the opioid receptors are not directly targeted by crotalphine, but rather lie somewhere downstream of its site of action. Bressan et al. (2016) have found that crotalphine acts as a selective partial agonist of TRPA1, strongly desensitizing the ion channel to both electrophilic and non-electrophilic agonists. This mechanism of action is essential for the analgesic effect of crotalphine. It has been speculated that the partial activation of TRPA1 by crotalphine increases the intracellular calcium concentration that in turn induces translocation of opioid receptors to the membrane, and hence increases the effectiveness of endogenous opioids [158]. These findings reveal that besides its therapeutic potential, crotalphine can serve as a novel tool to investigate the interaction between TRPA1 channel and the opioid system, and may lead to the development of new analgesic drugs that enhance opioid receptors activity without the typical side effects of opiates. 

Gsmtx-4, a toxin isolated from the venom of the Chilean rose tarantula (*Grammostola spatulata*), was found to potently activate TRPA1 [163]. However, it remains unclear whether this toxin activates TRPA1 by binding directly to the channel or by some other indirect mechanism of action. Previous studies revealed that Gsmtx-4 inhibits various mechanosensitive ion channels such as TRPC1, TRPC6, and Piezo1 [164,165,166]. This toxin acts by perturbing the outer and inner leaflet of the membrane causing a curvature of the membrane near the channel, which modulates the stretch-activated channel gating to favor the closed state [167,168]. Elucidating the precise mechanism in which Gsmtx-4 activates TRPA1 may provide us with insights regarding the role of TRPA1 in the mechanosensory pathway, which is still a controversial topic [169,170]. 

## 4. Acid-Sensing Ion Channels (ASICs)

Acid-sensing ion channels (ASICs) are a group of voltage-insensitive cation channels permeable mainly to sodium that are expressed in neurons of the pain pathway [22,172]. ASICs are activated by a decrease in the pH of the extracellular environment (ten-fold more sensitive than TRPV1). Thus, they are considered as primary sensors for acid [173,174]. Four distinct genes (ASIC1-4) encode the different ASIC channels. As ASIC1 and ASIC2 produce two functional splice variants (ASIC1a, ASIC1b, and ASIC2a, ASIC2b, respectively), a total of six ASIC subunits have been characterized so far [173]. ASIC subunits can assemble as heteromeric or homomeric trimers to produce a functional channel [22]. Each subunit consists of short intracellular N- and C-terminals, two transmembrane domains (TM1 and TM2), and a large extracellular domain [175]. Following the crystallization of chicken ASIC1a (cASIC1a), the structure of an individual subunit was depicted as a hand holding a ball, with the TMs representing a forearm, the junction between the TMs and the extracellular domain is regarded as a wrist and the extracellular domain forming palm, knuckle, finger, thumb, and b-ball domains [22,175,176]. While all ASICs share the same topology, they present different biophysical properties and expression profiles. In rodents, ASIC1, ASIC2, and ASIC3 are expressed in peripheral sensory neurons as they were detected in peripheral terminals and cell somas, but not in central terminals. ASIC1a and ASIC2 are mainly abundant in central neurons that receive, modulate, and process inputs from the periphery [22]. ASICs can also be found in non-neuronal cells, such as adipose cells, lung cells, and osteoclasts [174]. 

ASIC activation by pH produces and possibly sustains membrane depolarization that is sufficient to generate action potentials firing in nociceptors [22,177]. Tissue acidosis could occur in inflammation, trauma, tumors, ischemia, and following surgery [174]. Thus, ASICs could be physiologically activated in these instances. Additionally, proton activation of ASICs was implicated in gastritis, peptic ulceration, and other gastrointestinal-related pain pathologies, indicating these channels as potential targets for the relief of visceral pain [178,179]. In addition to protons, ASICs were shown to be modulated by endogenous molecules, synthetic compounds, and natural substances. ASIC activators include the endogenous agmatine and serotonin, which activate ASIC3-containing channels in inflammatory settings [22,180]. Additionally, ingredients of the inflammatory soup, including serotonin, ATP, and bradykinin through the PKC signaling pathway, indirectly modulate ASICs activity [174]. Other molecules implicated in inflammation like arachidonic acid and anandamide potentiate ASIC1, ASIC2, and ASIC3 towards protons [22,181]. Amiloride is a synthetic pore blocker that inhibits all ASICs, as well as other ion channels and exchangers [182]. Several nonsteroidal anti-inflammatory drugs (NSAIDs) including ibuprofen and diclofenac, were also found to directly inhibit specific ASIC channels, albeit with low potency (IC_50_ = 90–350 µM; COS and CHO cells) [22,183,184]. Nevertheless, ASIC inhibition might represent another route through which NSAIDs promote analgesia. In addition, anesthetics, both general (propofol) and local (lidocaine) were reported to have inhibitory effects on ASIC1a and ASIC3 [185,186]. Several molecules derived from plants that are in use for the treatment of pain in traditional medicine were shown to inhibit several ASICs as well [22]. However, while many small molecule ASIC modulators were discovered, their low potency and poor selectivity make them less than ideal probes in studying these channels. 

Growing interest in ASICs has led to extensive screening of venoms in search of new channel modulators. Indeed, several toxins that target ASICs were identified in venoms from spiders, sea anemones and snakes (Table 3). Due to their effectiveness and selectivity, these toxins represent an excellent and much needed pharmacological tool. While ASICs have diverse and complex pharmacology, toxins that modulate these channels provide a better understanding of their physiological and pathophysiological functions. These toxins were used to highlight ASICs that are important in several pain conditions and represent new approaches to pain management. Additionally, crystallization and structural modeling of toxin-bound ASICs, revealed different conformations of these channels as well as identified novel channel domains for pharmacological interventions. Furthermore, toxin-ASIC complexes could elucidate the pharmacophores of these toxins and validate their use as leads in drug development processes. Additionally, since they lack apparent toxicity in animal models, ASIC-targeting toxins themselves could have a therapeutic value.

The first ASIC-modulating toxin from an animal source that was described is Psalmotoxin1 (PcTx1). PcTx1 is a peptide extracted from the venom of the *Psalmopoeus cambridgei* spider [187]. This toxin has complex pharmacology, which varies between different species [173]. In addition, PcTx1 also presents distinct state-dependent activity on different ASICs [173]. This variability demonstrates that small changes in molecular interactions can have significant functional impacts on ASIC ligands. In rodents, PcTx1 is a potent inhibitor of ASIC1a and ASIC1a/ASIC2b channels (IC_50_ = 0.4–3.7 nM: *Xenopus laevis* oocytes) locking them in a desensitized state while it also stimulates ASIC1b [173,187,188,189,190]. It was suggested that the desensitizing effect stems from the toxin ability to increase ASIC affinity to protons thus rendering the channel desensitized in physiological pH and making PcTx1 effective in non-acidified tissues [174]. Nonetheless, PcTx1-cASIC1a complexes in different conformations (desensitized and two different open conformations: nonselective and Na^+^-selective) were crystallized in which the toxin was shown to bind in the interface of two subunits and interact with the channel’s pH sensor (acidic pocket) [191,192]. It was further suggested that PcTx1 mimics the binding of protons in this site [174]. While this toxin-channel interaction provided many insights regarding the structure and function of ASICs, it is not clear whether these crystallized structures could be used in drug design for humans, as PcTx1 presents species-specific effects and activates chicken ASIC1 [173]. Though acting as an agonist/antagonist in different settings, the net effect of PcTx1 injected intrathecally is analgesic in acute, neuropathic, and inflammatory pain models. Interestingly, it was suggested that this pain relief is met-enkephalin-dependent as PcTx1 inhibition of ASIC1a might facilitate the release of this opioid [193]. Additional studies are required in order to explore the possibility of engaging the opioid system with PcTx1 rather than using the deleterious opiates. Intrathecal PcTx1 was also found to produce visceral pain relief in colorectal distension [194]. In contrast, subcutaneous injections of PcTx1 do not possess any analgesic effect in acute and post-operative pain models [181,193,195]. This may suggest that in nociceptors, the toxin’s targets, ASIC1a homomers and ASIC1a/ASIC2a, are insignificant in these settings. Importantly, in vivo experiments with PcTx1 did not reveal any apparent adverse effects or acute toxicity [173]. Recently, a PcTx1 analog, named Hm3a, from the venom of the Togo starburst tarantula (*Heteroscodra maculata*) was characterized [196]. The two toxins present high identity and very similar pharmacological properties. However, Hm3a showed superior stability to PcTx1 in human serum, making it a more attractive tool in future studies [173,196]. Another PcTx1-related toxin is Hi1a from the Australian funnel-web spider (*Hadronyche infensa*) [197]. Hi1a is a bivalent inhibitory toxin comprised of two PcTx1-like ICK motifs connected by a short linker. However, unlike PcTx1, Hi1a partially inhibits ASIC1a and does not affect ASIC1b [197]. The very potent inhibition by this toxin (IC_50_ = 0.52 nM: *Xenopus laevis* oocytes) is also slowly reversible, reminiscent of the irreversible bivalent TRPV1 toxin, DkTx [69,197]. Thus, Hi1a represents the most selective and long-acting modulator of ASIC1a and an enticing new probe in investigating this channel role in nociception.

APETx2 from the venom of the sea anemone *Anthopleura elegantissima* presents inhibitory effect on rat and human ASIC3 homomers as well as several ASIC3 heteromers [198]. However, this peptide toxin was also shown to inhibit Na_V_1.8, Na_V_1.6, Na_V_1.2, and hERG channels, albeit with generally reduced potency (IC_50_ = 55 nM–2.6 μM: *Xenopus laevis* oocytes, rat DRG neurons; IC_50_ = 114 nM: *Xenopus laevis* oocytes; not specified; IC_50_ = 1.21 μM; *Xenopus laevis* oocytes, respectively) compared to its ASIC3 inhibition (IC_50_ = 63 nM; *Xenopus laevis* oocytes) [173,199,200]. Nevertheless, this lack of specificity could undermine the therapeutic value of this toxin. Molecular docking was used to elucidate APETx2 binding to ASIC3, suggesting the putative involvement of either the upper thumb or the wrist and palm domains in this association [201]. Further structural and mutagenesis studies have also suggested a pharmacophore for this toxin [199]. However, accurate characterization of APETx2 binding and its conformational implications are still lacking. Local injection of APETx2 induces potent analgesia in inflammatory pain, non-inflammatory muscular pain, and post-surgical pain models, demonstrating a role for ASIC3 in thermal and mechanical hyperalgesia [181,202,203]. APETx2 also reduces pain in an osteoarthritis model when given intra-articularly [203]. While ASIC3 was suggested to contribute to mechanical hypersensitivity in the colon, a potential analgesic effect of APETx2 also in this pain condition was not yet tested [178,204]. Overall, APETx2 highlights ASIC3 as a promising target for antagonists in pain management as it provides analgesia in somatic pain and potentially also in visceral pain. 

Mambalgins constitute a group of three ASIC inhibitory toxins derived from the venom of African black mamba (*Dendroaspis polylepis*; mambalgin-1 and mambalgin- 2) and the venom of the green Mamba (*Dendroaspis angusticeps*; mambalgin-3) [174,195]. The three toxins are highly homologous, differing by one amino acid from one another [174]. Indeed, mambalgins have the same pharmacological properties as they all inhibit rat and human ASIC1a and ASIC1b containing channels with high potency (IC_50_ = 11–252 nM: *Xenopus laevis* oocytes) [173,195]. Since mambalgins do not display mixed pharmacological effects, they were found to block ASIC-derived current to a greater extent in rat sensory neurons compared to PcTx1 [195]. Mambalgin-1 was suggested to bind to the closed state of ASIC1a and decrease the affinity to protons while only a partial pharmacophore of this toxin was presented [205,206]. In addition, mambalgin-2 was shown to bind to the acidic pocket [207]. Thus, while mambalgins present a three-finger toxin fold which greatly differs from the ICK scaffold of PcTx1 and Hi1a, the binding sites of these toxins may overlap substantially. However, co-crystallization of ASIC1 and mambalgins that will determine toxin-channel interactions is yet to be produced. Injecting mambalgins centrally produced analgesia in acute and inflammatory pain models in an opioid-independent manner, demonstrating that central ASIC inhibition can directly reduce pain [195]. This also highlighted the role of ASIC1a and ASIC2a in nociception as the ASIC1a/ASIC2a heteromer was necessary for achieving the pain relief effect [195]. Subcutaneous and intraplantar injections of mambalgin-1 also produced an analgesic effect, alleviating acute pain and thermal hyperalgesia [174,195]. This pain relief is ASIC1b-dependent, consistent with siRNA experiments silencing this subunit, demonstrating this subunit’s role in pain sensation [195].

An ASIC activating toxin, MitTx, was found in the venom of the Texas coral snake (*Micrurus tener tener*) [208]. This heterodimer toxin consists of two peptide subunits, MitTx-α (Kunitz-type) and MitTx-β (phospholipase A2-like), which are non-covalently bound [208]. MitTx activates in a pH-independent manner ASIC1a and ASIC1b homomers (IC_50_ = 9–23 nM: *Xenopus laevis* oocytes) in nanomolar concentrations as well as ASIC3 and ASIC1a/ASIC2a with lower potency (IC_50_ = 75–830 nM: *Xenopus laevis* oocytes) [208]. Additionally, this toxin potentiates ASIC2a pH response [208]. Importantly, MitTx was used in the crystallization of what is thought to be the first physiologically relevant cASIC1a open state structure that further elucidated the configuration of the channel’s selectivity filter [209]. In addition to the valuable insights into ASIC1a gating, this toxin-channel complex also revealed the MitTx binding site. It was found that in contrast to other toxins, MitTx binds to a single subunit with which it produces multiple interactions [209]. Pain response to intraplantar injection of MitTx is largely associated with ASIC1. In accordance, MitTx-evoked depolarization in rat trigeminal (TG) neurons was shown to be mainly ASIC1 subunit dependent thus demonstrating the role of this subunit in peripheral nociception [208]. However, while ASIC1 channels activated by protons undergo rapid inactivation, MitTx induced activation is persistent [174]. As ASIC1a channels are also permeable to Ca^2+^, achieving analgesia following a robust activation of these channels might be possible similarly to what happens in capsaicin treatments [172]. However, neuronal degeneration and desensitization following the initially produced pain were not reported so far in MitTx applications. Furthermore, the available ASIC inhibitory toxins, which seem to be well tolerated, present leads that are more viable in the development of ASIC-targeting analgesics.

## 5. Voltage-Gated Sodium Channels

Voltage-gated sodium channels (Na_V_) are complex transmembrane proteins that play an important role in the generation of action potentials in excitable cells. This family of sodium channels includes nine known members named Na_V_1.1–1.9. Na_V_ channels are comprised of one long α subunit that consists of four homologous domains (domains I–IV). Each domain contains six transmembrane segments in which the first four (S1–S4) form the voltage sensor while segments S5–S6 form the ion-conducting pore. The membrane potential regulates activation of these channels as depolarization triggers conformational changes, which in turn lead to a rapid influx of Na^+^ ions into the cell through the channel’s pore. Na_V_ channels are distributed in electrically excitable cells where they play a critical function in the initiation and propagation of action potentials [212,213]. The different Na_V_ isoforms present distinct expression profiles and are associated with different functional properties in the corresponding tissues. Na_V_1.1, Na_V_1.2, and Na_V_1.6 are abundantly expressed in the central nervous system (CNS), whereas Na_V_1.4 and Na_V_1.5 are predominantly expressed on skeletal and cardiac myocytes, respectively. Na_V_1.3 channels are mostly present during embryonic development; however, it has been found that the expression levels of these channels increase significantly in the dorsal root ganglion (DRG) after peripheral nerve injury. In the peripheral nervous system (PNS), sensory neurons express multiple Na_V_ channel subtypes while the dominant isoforms are Na_V_1.7, Na_V_1.8, and Na_V_1.9 [214,215,216]. Numerous findings indicate that Na_V_1.7, Na_V_1.8, and Na_V_1.9 are significantly important for the transmission of painful stimuli [24,217,218,219,220]. Indeed, loss-of-function mutations of Na_V_1.7 have been linked to complete insensitivity to pain [221,222,223]. This congenital insensitivity to pain (CIP) is a rare genetic disorder characterized by the inability to sense acute and chronic pain while all other functions are normal. CIP patients are prone to life-threatening injuries including self-mutilation, repeated burns, and bone fractures [224]. Accordingly, gain-of-function mutations of Na_V_1.7 channel have been linked to several painful disorders, including inherited erythromelalgia (IEM) and paroxysmal extreme pain disorder (PEPD) [225,226,227]. IEM and PEPD are rare disorders characterized by episodes of severe burning pain sensation that most commonly occurs in distal extremities and the perirectal region, respectively [224]. Gain-of-function mutations of Na_V_1.7 have also been linked to small fiber neuropathy (SFN). SFN is characterized by a dysfunction of peripheral small diameter myelinated (Aδ) and unmyelinated (C) nerve fibers which results in a variety of symptoms, including neuropathic pain and autonomic neuropathy [228,229]. More recently, gain-of-function mutations in Na_V_1.8 and Na_V_1.9 have also been identified in SFN patients indicating that mutations in these channels contribute to the pathophysiology of painful peripheral neuropathy [230,231,232,233]. In agreement with these findings, local treatments with non-selective Na_V_ channel blockers, such as lidocaine, have been shown to attenuate acute, inflammatory, and neuropathic pain [217,234,235,236]. Additionally, previous studies have shown that Na_V_1.8 and Na_V_1.9 subtypes crucially and specifically involved in visceral nociception [237,238,239]. For instance, knockout of Na_V_1.8 or Na_V_1.9 in rodents reduced visceral pain and hyperalgesia, which emphasize the role of these isoforms in gastrointestinal disorders [240,241,242]. Overall, these studies indicate that Na_V_1.7, Na_V_1.8, and Na_V_1.9 have key roles in nociception and pain sensations. Moreover, several studies have demonstrated that Na_V_1.1, Na_V_1.3, and Na_V_1.6 also play important functions in pain [243,244,245,246,247,248,249]. Further research is needed in order to elucidate precisely the link between specific Na_V_ channel isoforms and various pain conditions. Taken together, the development of novel subtype-selective agents could have great therapeutic potential for treating a wide spectrum of pain conditions. These compounds could be potentially used as safer alternatives with regard to opioids for long-term pain management.

Interestingly, numerous peptide toxins isolated from venoms of various animal species including fish, scorpions, spiders, sea anemones, and cone snails have been found to interact with specific Na_V_ channel isoforms (Table 4) [250,251,252,253]. By producing toxins that target Na_V_ channels, venomous animals are able to efficiently paralyze prey or defend against predators highlighting the significance of the Na_V_ channels in electrical signaling and neuromuscular function. These toxins act on at least six different characterized binding sites (Toxin sites 1−6) which inhibit, activate, or modulate the gating properties of Na_V_ channels [254,255]. The interaction between the toxin peptides and Na_V_ channels occur through two distinct mechanisms: either by blocking the channel pore (Toxin site 1) or by modifying channel gating (Toxin sites 2-6). Pore-blocking toxins bind at site 1 and physically block the flow of Na^+^ ions through the channel, thereby preventing the generation and propagation of action potentials. A notable member of this class is tetrodotoxin (TTX), a potent Na_V_ channel blocker found in many pufferfish species. This toxin blocks the sodium influx by binding to the outer vestibule of the channel pore, which results in complete inhibition of the channel [256,257]. 

Unlike the pore-blocking toxins, the gating modifier toxins modulate Na_V_ channels by binding to the voltage sensor-related domains (segments S1–S4 and their extracellular linkers). Interestingly, previous works have identified at least five distinct binding sites on Na_V_ channels for various gating-modifier toxins [255,258,259,260]. In general, these toxins either activate or inhibit the Na_V_ channels by altering the movements of the voltage sensor, trapping the channel in a specific conformation. Gating-modifier toxins affect the activity of the channel in several mechanisms. For instance, scorpion α-toxins and different sea anemone toxins bind at toxin site 3 inducing slower or diminished channel inactivation, which, in turn, leads to prolonged action potentials. These toxins specifically bind to the extracellular S3–S4 loop in domain IV and hold the transmembrane segment in its inward position, thereby blocking the channel’s fast inactivation [250,261,262,263]. On the other hand, scorpion β toxins enhance activation of Na_V_ channels by binding at toxins site 4 and hold the voltage sensor in domain II in its outward-activated position [264,265,266]. Upon binding, scorpion β-toxins shift the voltage dependence of activation towards more negative potentials increasing the probability of channel opening. The existence of distinct toxin binding sites and different mechanisms of action could potentially lead to the development of new Na_V_ modulators with diverse therapeutic effects. This underlines the importance of identifying the precise various binding sites on the Na_V_ channel. 

Preclinical and clinical studies have shown promising analgesic effects of several animal toxins targeting Na_V_ channels [252]. One of those promising candidates is TTX [267,268]. Na_V_ channels are generally classified as TTX-sensitive (Na_V_ 1.1–Na_V_ 1.4, Na_V_ 1.6, and Na_V_ 1.7) or TTX-resistant (Na_V_ 1.5, Na_V_ 1.8, and Na_V_ 1.9) according to the binding affinity to this toxin. The partial isoform selectivity of the toxin could be exploited at the drug design and therapeutic levels. The development of drugs that selectively inhibit the activity of the peripheral Na_V_ channels involved in nociception could potentially reduce the adverse effects associated with non-selective Na_V_ channel blockers [269,270]. While TTX is not selective towards pain-related Na_V_ channels, this toxin does not act centrally as it has only a minimal ability to cross the blood-brain barrier. Indeed, several studies performed in humans and animals have shown that TTX is a potential analgesic compound administration of TTX reduced pain-related behaviours in several rodent models of inflammatory and neuropathic pain without any adverse side effects [271,272,273,274,275]. Moreover, subcutaneous injections of TTX attenuated visceral pain and reduced capsaicin-induced mechanical hyperalgesia in mice models of visceral pain [67,276]. Currently, TTX is under clinical investigation for the treatment of neuropathic and cancer-related pain [277,278]. TTX effectiveness together with its reduced risk of CNS-related side effects further emphasize the potential of selective peripheral Na_V_ channel blockers to serve as therapeutic agents in pain and inflammatory conditions. Nonetheless, the search for highly selective inhibitors, which act on one or very few Na_V_ channel isoforms, is an ongoing process. However, the development of truly isoform-selective inhibitors is extremely challenging [279]. 

One of the first and the most studied selective Na_V_ 1.7 inhibitors is ProTx-II. This gating-modifier toxin isolated from the venom of the Peruvian green velvet tarantula (*Thrixopelma pruriens*) shifts the voltage dependence of activation towards more positive potentials by trapping the voltage sensor in the closed state [146,280,281]. However, the precise binding site of ProTx-II was not completely elucidated [280,282,283]. While ProTx-II inhibits multiple sodium channel isoforms it has been reported to be ~100-fold more selective towards inhibition of Na_V_ 1.7 over other Na_V_ channels [146,282,283]. The selectivity of ProTx-II for Na_V_ 1.7 makes it an attractive lead compound for the development of new antinociceptive drugs. However, in vivo studies of ProTX-II yielded inconclusive results. While some groups showed that ProTX-II indeed exerts analgesia with no significant effect on motor function, another group reported that injections of ProTx-II failed to elicit pain relief in rodent models of acute and inflammatory pain [282,284,285]. It has been suggested that the observed differences are due to a narrow therapeutic window of the toxin. ProTx-II was found to be lethal in rats probably by off-target inhibition of Na_V_ 1.5 and Na_V_ 1.6, which are critical for cardiac activity and action potential generation in motor neurons, respectively. This suggests that Na_V_ 1.7 channel blockers must be extremely selective in order to avoid serious adverse side effects. By using ProTX-II as a scaffold, a new highly selective and potent Na_V_ 1.7 inhibitor (JNJ63955918; IC_50_~10nM in HEK293) was engineered. This peptide produces analgesia and insensitivity to pain resembling the 1.7-null phenotype observed in human and mice without causing any detectable adverse effects [285].

β-TRTX-Gr1b is a toxin from the venom of the Chilean rose tarantula (*Grammostola rosea*) that shares ~90% sequence similarity with ProTX-II [286,287,288]. When injected in rats, β-TRTX-Gr1b produced analgesic effects in several pain models without any confounding side effects [287,289]. These studies demonstrate that new selective blockers of Na_V_ 1.7 could be identified in other venoms or rationally designed and optimized based on sequence homology [290,291,292,293]. Indeed, a plethora of toxin peptides targeting the Na_V_ 1.7 channel subtype has been characterized in various animal species [294,295,296,297,298,299,300]. These findings could potentially lead to significant advancement in the discovery of Na_V_ 1.7-based analgesics. One interesting peptide is the μ-theraphotoxin-Pn3a toxin, isolated from the venom of the tarantula *Pamphobeteus nigricolor*. This toxin is a highly selective inhibitor of Na_V_1.7. While μ-theraphotoxin-Pn3a solely injected to mice has no analgesic effect, when it is administrated with sub-therapeutic doses of opioids this peptide produces profound pain relief [299]. This raises the possibility of a crosstalk between Na_V_ channels and the opioid system. This peptide toxin may act as an amplifier that increases the potency or the efficacy of opiates. Thus, such molecules may have the potential to reduce the use of opiates. Although Na_V_1.7 is one of the most promising targets for the treatment of pain, toxins targeting other Na_V_ channel isoforms have also been found as promising leads for the development of new analgesic drugs [252,253,301,302,303]. For instance, μO-conotoxin MrVIB from *Conus marmoreus* selectively inhibits the Na_V_1.8 subtype and has analgesic properties in chronic pain models without affecting motor functions [301,302]. The effects of MrVIB reveal that selective Na_V_ 1.8 blockers can be used in neuropathic pain conditions with a greater therapeutic index than non-selective inhibitors such as lignocaine. Mutagenesis studies have shown that MrVIB exerts its activity by binding to the pore loop in domain II of Na_V_1.8 [304,305]. Overall, MrVIB indicate Na_V_1.8 as a promising target for the treatment of chronic pain.

δ-theraphotoxin-Hm1a (Hm1a) and δ-theraphotoxin-Hm1b (Hm1b) are two toxins from the venom of the Togo Starburst tarantula (*Heteroscodra maculata*) which were found to activate Na_V_1.1 channels selectively [245]. Hm1a and Hm1b elicit pain and mechanical hypersensitivity in mice, revealing an unexpected role for Na_V_1.1 channels in mediating mechanical pain. Activation of Na_V_1.1 by Hm1a evokes robust pain behaviors and mechanical allodynia without triggering neurogenic inflammation. These findings demonstrate that inhibition of Na_V_1.1 may represent a new and novel therapeutic strategy for treating pain. It was also found that Hm1a inhibits the fast inactivation of Na_V_1.1 by binding to the S3b–S4 and S1–S2 loops in domain IV. Interestingly, the subtype selectivity of Hm1a mainly depends on the S1-S2 loop in domain IV, providing a potential strategy for designing other subtype-specific ligands. It appears that the variability in the S1–S2 voltage-sensor region between the different isoforms may be critical for selective subtype modulation of Na_V_ channels [245,306]. The high selectivity profile of Hm1a has been exploited to investigate the gating mechanisms of the Na_V_1.1 channel. Although inhibition of Na_V_1.1 is a promising approach for treating pain, it has been suggested that activation of this channels may hold therapeutic potential for disorders such as epilepsy, Alzheimer’s disease, and schizophrenia [307]. A better understanding of the binding mechanisms of Hm1a may lead to the development of new compounds with similar selectivity and functional profiles that could be promising lead drug candidates for the treatment of CNS-related diseases. Furthermore, the role of Na_V_1.6 channel in stretch-sensitive colorectal afferent endings has been studied using several animal toxins. The selective Na_V_1.6 antagonists μ-conotoxin GIIIa, μ-conotoxin PIIIa, or tetrodotoxin significantly attenuated afferent responses to stretch, while selective Na_V_1.8 and Na_V_1.7 inhibitors have no significant effect on afferent responses to stretch. This shows that Na_V_1.6 contributes significantly to the tonic firing of stretch-sensitive colorectal afferent endings highlighting its role in visceral pain [308]. Overall, these findings demonstrate that besides their potential as therapeutic agents, subtype-selective toxins provide excellent and unique tools to study the role of each Na_V_ channel isoform in various pain conditions.

## 6. Discussion

Pain is a complex health concern affecting millions of people worldwide. Ineffective pain management has a significant impact on the patient’s quality of life, consequently posing a considerable challenge to society. Despite excessive research over the past decades, the mechanisms underlying the transition of acute to chronic pain remains unclear. Nowadays, the most effective drugs for treating many pain syndromes are opioids. However, opioid use is associated with multiple adverse effects in addition to tolerance, physiological dependence, addiction, and abuse. Indeed, the rapid increase in the use of prescription opioid drugs in the United States is well correlated with the increasing opioid overdose death rates. The misuse and addiction to opioids is a severe crisis that has devastating consequences on public health and economy. Unfortunately, currently there are no effective alternatives to replace opioids. Therefore, new innovative approaches are required in order to develop non-opioid alternatives for managing chronic pain [309,310].

Animal venoms are an outstanding source of biologically active toxins with diverse targets and functions [311,312]. Venoms are a mixture of small molecules and peptides that act selectively on their respective targets to exert their effect. Thus, isolation and identification of the different venom components could potentially be exploited for the development of new therapeutic compounds. Indeed, several toxin-based drugs have been approved by the FDA for treating various diseases, and many more are currently under pre-clinical and clinical investigation [313,314]. In addition to their therapeutic potential, toxins are excellent tools to investigate channels’ structure. Toxins play an important function in revealing specific conformations of multiple ion channels by trapping the channel in a particular state that allows crystallization. Moreover, many toxins have been utilized to study the molecular mechanisms underlying channel gating. For instance, diverse gating mechanisms of voltage-gated sodium channels have been identified by using toxins that selectively interact with these channels. This demonstrates that toxins are tremendously useful biochemical tools that, indeed, advanced our understanding of fundamental biological processes [25,315,316].

The mammalian pain pathway is equipped with an array of unique receptors that enable it to detect and react in a timely manner to a variety of stimuli. Venomous animals exploit these receptors to evoke pain for offensive or defensive purposes. Animal venoms contain various factors that selectively activate pain-related receptors and ion channels, including TRPV1, TRPA1, ASICs, and voltage-gated ion channels [25]. Additionally, these venoms contain a host of inhibitory toxins, which were shown to be highly specific and potent. The discovery of highly selective inhibitory toxins opens a new promising approach to pain therapy. Numerous lines of evidence have demonstrated the therapeutic potential of these inhibitory toxins that can induce strong analgesic and anti-inflammatory effects in various animal models of pain. Furthermore, several toxin peptides are currently under clinical investigation for the treatment of several pain conditions [311,312,317]. Although inhibition of pain-related receptors appears to be the most logical therapeutic strategy, several findings have demonstrated that prolonged activation of these receptors could produce profound analgesic effects, probably via desensitization of these receptors and defunctionalization of nociceptors. For example, topical treatments of capsaicin can promote pain relief in certain pain conditions. It appears that both agonists and antagonists are promising drug candidates. It was further speculated that inhibitory peptides might provide immediate pain relief, while agonists may exert long-lasting analgesia through desensitization mechanisms.

Isolating the different components participating in pain perception is a major challenge in understanding pain. As such, venom peptides targeting specific pain receptors offer a unique and novel approach to investigate the roles of the different receptors in nociception and analgesia. Indeed, toxins have been instrumental in analyzing the mechanisms that underlie both somatic pain and the less-studied visceral pain. Adequate understanding of pain and its mechanisms may facilitate the development of more effective therapeutic strategies. As mentioned above, there is a need for the development of safer alternatives to opioids for pain management. Therefore, identification of peptides that selectively modulate peripheral pain receptors without disrupting other ion channels, especially in the central nervous system, may reduce the adverse effects associated with opioid treatment.

Animal venoms are a complex mixture of a variety of biological substances. However, the dominant components of most venoms are peptides [311,312]. One of the major drawbacks in translating peptides into clinically useful therapies is their low bioavailability [318]. When administered orally, peptide-based drugs are susceptible to rapid digestion by proteolytic enzymes in the gastrointestinal tract. Moreover, the ability of peptides to cross physiological barriers and membranes is limited. Due to their low bioavailability, peptides need to be delivered via injection, which results in low patient compliance and may require skilled healthcare providers. In addition to the lack of adequate oral bioavailability, high production cost and low storage stability are considerable challenges in industrial production of peptides. Several strategies have been developed to overcome these drawbacks [319,320,321]. In general, various chemical modifications of peptides improve their stability and oral bioavailability markedly. Common modifications of peptides include cyclization, methylation, and substitution of specific amino acids [322,323,324,325]. Second, encapsulation of peptides into drug delivery systems provides a novel strategy to protect them from enzymatic degradation and to control their release [326,327,328]. Additionally, conjugation of peptides to polymer chains, oligosaccharides, or fatty acids prevents the fast renal clearance of the peptides [329,330]. Improving the bioavailability of the peptides at the site of action can significantly reduce drug loads and adverse side effects. These strategies offer unique approaches for oral delivery of peptides that may lead to further development of peptide-based drugs with great therapeutic potentials. Of note, as mentioned above, several toxins consist of ICK motifs. This motif is very stable in different pHs and resist proteases. Thus, ICK toxins may require less modification for therapeutic use.

The repertoire of unique peptides derived from venoms of various animal species is enormous. Some venoms contain thousands of unique peptides yielding an impressive reservoir of millions of bioactive peptides [311,312]. Given their high potency and selectivity, venom peptides have attracted considerable interest in the development of new analgesic drugs. However, despite recent progress, only a small fraction of venom peptides have been characterized and experimentally analyzed. Increasing efforts to identify unique toxin peptides will considerably advance our understanding of the mechanisms underlying pain and may provide novel opportunities for developing more effective life-changing treatments.

## Figures and Tables

**Table 1:** Toxins targeting TRPV1.

Toxin	Nociceptive Effect	Pain Model
Capsaicin *Capsicum* family	Analgesia (following pain)	Acute (rats) [56,92,93]. Neuropathic pain (humans) [94,95,96,97,98].
RTX *Euphorbia resinifera*	Analgesia (following pain)	Acute (pigs, mice and rats) [99,100,101,102]. Inflammatory (dogs, rats and mice) [65,101,102]. Cancer-related pain (humans and dogs) [65,103].
VaTx1-3 *Psalmopoeus cambridgei*	Pain	Acute (mice) [68].
DkTx *Ornithoctonus huwena*	NA	-
RhTx *Scolopendra subspinipes mutilans*	Pain	Acute (mice) [75].
BmP01 *Mesobuthus martensii*	Pain	Acute (mice) [76].
APHC1,3 *Heteractis crispa*	Analgesia	Acute (mice) [55]. Inflammatory (mice) [55]
AG489 *Agelenopsis aperta*	NA	-

**Table 2:** Toxins targeting TRPA1.

Toxin	Nociceptive Effect	Pain Model
ProTx-I *Thrixopelma pruriens*	NA	-
Phα1β *Phoneutria nigriventer*	Analgesia	Acute [148,150]. Inflammatory (mice) [148,150,153]. Neuropathic (rats) [148,150,153,171]. Post-operative pain (mice) [149]. Cancer-related pain (mice and rats) [151,152,153].
Ms 9a-1 *metridium senile*	Analgesia	Acute and inflammatory (mice) [144].
Ueq 12-1 *metridium senile*	Analgesia	Acute and inflammatory (mice) [144,157].
Crotalphine *Crotalus durissus terrificus*	Analgesia	Acute (mice) [161]. Inflammatory (rats) [158,159,161]. Neuropathic (rats) [160]. Cancer-related pain (rats) [162].
Gsmtx-4 *Grammostola spatulata*	NA	-

**Table 3:** Toxins targeting ASICs channels.

Toxin	Nociceptive Effect	Pain Model
PcTx1 *Psalmopoeus cambridgei*	Analgesia	Acute (mice) [193]. Inflammatory (mice) [193]. Neuropathic (mice and rats) [193]. Visceral (rats) [194].
Hi1a *Hadronyche infensa*	NA	-
Hm3a *Heteroscodra maculata*	NA	-
APETx2 *Anthopleura elegantissima*	Analgesia	Inflammatory (rats) [181,210,211]. Post-operative pain, rats [202].
Mambalgin1-3 *Dendroaspis polylepis* *Dendroaspis angusticeps*	Analgesia	Acute (mice) [195]. Inflammatory (mice) [195].
MitTx *Micrurus tener tener*	Pain	Acute (mice) [208].

**Table 4:** Toxins targeting voltage-gated sodium channels.

Toxin	Nociceptive Effect	Pain Model
Tetrodotoxin *Tetraodontidae*	Analgesia	Inflammatory (mice and rats) [273,274,276]. Neuropathic (mice and rats) [271,273,275,276]. Visceral (mice and rats) [67,276]. Cancer-related pain (mice and humans) [272,273,277,278].
ProTx-II *Thrixopelma pruriens*	Analgesia	Acute and inflammatory (rats) [285]. Diabetic neuropathic pain (mice) [284].
β-TRTX-Gr1b *Grammostola rosea*	Analgesia	Acute and inflammatory (rats) [289].
μ-theraphotoxin-Pn3a *Pamphobeteus nigricolor*	Analgesia(only when co-administrated with opioids)	Acute and inflammatory (mice and rats) [299].
μO-conotoxin MrVIB *Conus marmoreus*	Analgesia	Acute (rats) [301]. Inflammatory (rats) [302]. Post-operative pain (rats) [301]. Neuropathic (rats) [302].
δ-theraphotoxin-Hm1a δ-theraphotoxin-Hm1b *Heteroscodra maculata*	Pain	Pain and mechanical hypersensitivity (mice) [245].
